# Proceedings Medical Association of Arkansas

**Published:** 1874-01

**Authors:** 


Proceedings of the Medical Association of the State of Arkansas; Little Rock, 1873.—The Association convened on the 9th of October, Dr. D. A. Linthicum, President, in the chair.

In looking over the daily proceedings we observe this Association has not been more fortunate than our other State Medical bodies, in keeping out of its meetings matters of personal controversy among members. The Arkansas Association, however, very wisely dispensed with the Committee on Medical Ethics, and established a "Judicial Council," consisting of six members, two of whom are to be annually elected; and have invested this Council with full power "to consider all ethical subjects, all questions of a personal character or controversy, including complaints and protests;" "and its decisions shall be *final* and *binding* upon all parties."

The annual address of the President is replete with advice, counsel and exhortation suited to the wants of his auditory. We are tempted to extract for our readers the following closing paragraphs of this address:

"I would most earnestly recommend you to be charitable to yourselves. There is a certain old maxim, and a very true one, that 'charity begins at home.' In your pursuit after science you may lose sight of those things that are necessary for self-preservation, and contract a disease that is very peculiar to our profession, the name of which you will not find in your classification, but which I will denominate *poverty*, and we may very naturally infer from the name that it is very troublesome, especially in the chill and winter months of declining life. I am an advocate for collecting what is our due, from those who are able to pay, and then taking care of it. Those whom we serve will not take care of us when we can no longer serve them. We are for use, not for ornament. I only speak thus to show you that when we can no longer work, we will soon be forgotten, and onr places quickly filled by others; and it will be well for us, after we have threaded the toilsome way of the medical profession until the oil in the lamp of life has burned low, and nerve-force wellnigh exhausted, and the weight of a straw becomes a burden to our enfeebled bodies, if we are not dependent on the charities of our former patrons. Better, far better, to be so girded about with affluence as to be shielded from the whims and dictations of selfish and unfeeling men, when the infirmities of age bid us cease our conflict with the world.

"Above all things, I beg of you to maintain peace, good-will, and brotherly love in your midst. Dissension and discord should be frowned down and driven out, should they dare to enter our sacred haunts. Our lease of life is too short for quarreling, and friendship too precious to be cast away. How very miserable would our lots be, with no friends to love and sympathize with us. No tender hands to close our eyes, when this world is receding from our view and our sight becomes forever dim; no one to shed a tear and mourn for us in our last moments, or to twine a wreath of flowers over the spot where we lay down to our last sleep.

"In conclusion, I would say to you, that while in the midst of life prepare for death. The blade of grass, the dew-drop, the budding rose, the lightning's flash, the systole and diastole of the human heart, the thrilling of the delicate nerve, the paling and coloring of the cheek, all speak to us of a great first cause. There is a divinity that stirs within us; the heavens themselves point out our hereafter, and intimate eternity to man. In the face, then, of the overpowering testimony that is daily presented us, conclusive of the immortality of the soul, let us so demean ourselves as to be prepared when the hour cometh that the sentinel who holds the keys to the celestial city shall throw aside the gates and bid the grand army of philanthropists enter upon that rest and joy prepared by God for the faithful laborers in His earthly vineyard."

The Special Judicial Committee close their report upon the Hot Springs' controversy by suggesting:

"Tenth. That, in order to maintain peace and harmony in the future, the past must be buried, and the future characterized by charity for each other's faults and professional pride; and a sincere love of science must be *positively paramount* to all else when assembled as the State Medical Association."

The pamphlet contains the report by Dr. W. H. Hawkins of a case of *Puerperal Mania, successfully treated with chloral.* "The chloral was used in this case eighty days, with perfect safety to the fœtus and a complete restoration of the mother, both mentally and physically." "Nearly eight pounds of chloroform was administered in this case, where its effects produced, latterly, insomnia, restlessness and cerebral excitement; nearly two pounds solid hydrate of chloral was taken with the effect of procuring sleep quiet and rest, with a restoration to health."

Dr. J. A. Dibrell's report of a case of *Contagion of Secondary Syphilis*, and *Vital Statistics of Little Rock, Ark.*, by Dr. R. G. Jennings, follow. Dr. J. remarks that "consumption is making rapid strides among the colored race;" and attributes the change to improvidence and the loss of the fostering care of their former owners. He combats the idea that residence in malarial regions tends to counteract or retard the development of tubercle; and expresses the opinion: "Miasmatic poison vitiates the condition of the system to such an extent as, undoubtedly, to favor tuberculosis when there already exists a predisposition in this direction." "In a period of twenty-five months, Little Rock has recorded eleven deaths from gun-shot wounds and injuries, four drowned, and five deaths from poisons, making a total of thirty-six deaths from violence—a very large ratio, indeed."

The number of *still-births* is seventy-nine, of which twenty were white and fifty-two colored. "Here we again observe that the mortality of the colored in still-births largely exceeds that of the white."

The President for 1874 is Dr. E. R. Duvall, of Fort Smith, and the Corresponding Secretary, Dr. P. R. Ford, of Helena. The next meeting of the Association takes place in Pine Bluff on the second Monday in October, 1874.

